# Forecasting success via early adoptions analysis: A data-driven study

**DOI:** 10.1371/journal.pone.0189096

**Published:** 2017-12-07

**Authors:** Giulio Rossetti, Letizia Milli, Fosca Giannotti, Dino Pedreschi

**Affiliations:** 1 Knowledge Discovery and Data Mining Laboratory, ISTI-CNR, Pisa, Italy; 2 Computer Science Department, University of Pisa, Pisa, Italy; East China Normal University, CHINA

## Abstract

Innovations are continuously launched over markets, such as new products over the retail market or new artists over the music scene. Some innovations become a success; others don’t. Forecasting which innovations will succeed at the beginning of their lifecycle is hard. In this paper, we provide a data-driven, large-scale account of the existence of a special niche among early adopters, individuals that consistently tend to adopt successful innovations *before* they reach success: we will call them *Hit-Savvy*. *Hit-Savvy* can be discovered in very different markets and retain over time their ability to anticipate the success of innovations. As our second contribution, we devise a predictive analytical process, exploiting *Hit-Savvy* as signals, which achieves high accuracy in the early-stage prediction of successful innovations, far beyond the reach of state-of-the-art time series forecasting models. Indeed, our findings and predictive model can be fruitfully used to support marketing strategies and product placement.

## 1 Introduction

Every day, new artists appear on the music scene, new products are launched onto retail markets, new restaurants and businesses open up. Every day, people make choices: which artists to listen to, which items to buy at the supermarket, which restaurants to visit. Consumers’ choices determine which *innovations* (artists, products, businesses) will reach success and achieve large diffusion, and which ones will not. To reach success, innovations need to reach/target the right *adopters*. Several classical studies [1–3] analyzed the different phases of product’s lifecycle, from the first appearance on the market to vanishing. Rogers [4] described a peculiar family of adopters: the *innovators*, e.g., the ones that adopt an innovation before it becomes mainstream, the ones that do not need peer pressure to make their choice. From a different, psychological perspective, Tetlock et al. [5, 6] recently identified another exciting niche of individuals, called *super-forecasters*, that continuously make correct predictions of future events (in controlled Q&A experiments). Tetlock’s study, which ran over a number of years, aimed to understand whether people could predict an explicit yes/no time-limited question. To make predictions, the *forecasters* were explicitly allowed to prepare themselves researching the particular topics they were asked about. Moreover, forecasters were allowed to change their predictions as time goes by to match their evolving feelings about the outcome as the deadline for the question grew closer. Citing [6], *super-forecasters* are

*“[..] people whose analytic ability is considerably better than random (or who, in financial analyst terms*, *“beat the market”)”*

namely, all those individuals able to provide the correct answers on a regular basis, thus making predictions having a precision far above the average.

In this work we address a question at the intersection of the two lines above: are there innovators with *passive* super-forecaster abilities? Or equivalently, are there users that consistently adopt, before others, innovations that will later reach success? To answer such questions, we adopt a data-driven approach evaluated on two real datasets of supermarket transactions and musical listenings.

Differently, from Tetlock’s approach, we do not ask users to express their forecast whether an innovation will be a success; we observe what and when they adopt (buy or listen) in the recorded transactions. Indeed, the niche of users we target—we call them *Hit-Savvy*—do not train themselves to produce a correct guess. Conversely, they regularly chose to adopt innovations (we will refer to them as *“items”*) that are likely to reach success in the future. Such difference is profound: adoption choices of *Hit-Savvy* are not driven by the desired outcome as for Tetlock’s *super-forecasters* (i.e., early-adopters who listen to novel music do not necessarily do so because they think the artist will be successful) but by personal taste.

The first contribution of our study, described in Section 2, is the discovery that *Hit-Savvy* *do exist* and that their peculiar behaviour *perdure* through time. We empirically observe in our data a niche of innovators that exhibit a surprising propensity to adopt future successful artists and products prevalently: moreover, such *Hit-Savvy* tend to last in time, retaining their ability for months or even years.

Moving from such results we addressed a question whose answer can deeply impact marketing strategies: can *Hit-Savvy* be used to *predict* whether an innovation in the early stage of its lifecycle will reach success in the future? Our second contribution, illustrated in Section 3, answer such question by describing a predictive analytical process that, using *Hit-Savvy* as signals, achieves high accuracy in the early-stage prediction of successful innovations. In Section 4, we validate our method on the previously analyzed datasets observing high precision and recall of successful *items* prediction, far beyond the state-of-the-art models based on time series forecasting.

We believe that our data-driven study sheds a new light at the intersection of two increasingly important themes, discussed in Section 5: the *diffusion of innovations* and the *science of success*. Finally, Section 6 concludes the paper introducing future directions of research.

## 2 Successful items and their adopters

*Super-forecasters* are ubiquitous still not easy to spot. Given a set of questions regarding future events each one of us, specialist or not can make his prediction: however, only a handful of people will be able to correctly guess the future more than the average. This peculiar minority, driven by chance or intuition, is strictly tied to the type of questions asked and to the definition we adopt to identify successful answers. In the classic scenario offered by Tetlock’s work [5], successful answers are those that capture the exact outcome of an event (e.g., the fact that a particular conflict ended, or that a bill has been approved by the Congress), while *super-forecasters* are those individuals able to correctly guess such outcome before it happened.

In our scenario, however, the valuable individuals we are searching for possess different characteristics w.r.t. Tetlock’s *super-forecasters*, and are subject to different constraints as well:

- Firstly, the niche of individuals we target do not actively train themselves to produce predictions but, by chance or luck, are constantly involved in the adoption of *successful items*;
- Secondly, conversely from *super-forecasters*, to be *Hit-Savvy* it is not sufficient to correctly *“guess”* the future but it is mandatory to do so before others do.

Moreover, if in Tetlock’s experiment each question has one and only correct answer (yes or no) in our settings the success of an artist, a product, as well as an idea, can be measured in multiple ways (i.e. considering a musician it can be the number of disks sold, the volume of his fanbase as well as how long took for his name to became easily recognizable from the public).

We can say that to identify *Hit-Savvy* we need to fix at least two domain-specific notions: (i) what it means for an *item* to be a successful one, and (ii) who are the *innovators*, i.e., those users that adopt an item before others do.

In this section, firstly we introduce the datasets we used in our experimental analysis, 2.1, then we propose two alternative definitions of success, 2.2. Finally, we describe a methodology tailored to identify *Hit-Savvy*, 2.3, and briefly discuss some of their properties, 2.4.

### 2.1 Experimental data

This paper describes the analytical results of a data-driven investigation, for this reason as a first step it is mandatory to introduce the datasets analyzed.

- **Last.fm**: Last.fm (http://www.lastfm.com) is an online social network platform where users can share their music tastes and discover new artists and genres. The crawled dataset is composed of two years (2010-2012) of listenings for 70K UK users: we considered only novel artists having at least 500 listenings. The data, involving users weekly artist listening charts, were collected in compliance with Last.fm API Terms Of Service (https://www.last.fm/api/tos).
- **Coop**: Market Basket Data from the largest Italian retail Company; our dataset span over a year (Jan-Dec 2011) and is extracted from a seven years long itemised timestamped transaction records. We considered only novel products launched into the market within such temporal window, e.g., those for which no transaction records were present in the complete dataset until the selected period.

All the user related information of both datasets—namely, Last.fm and Coop user identifiers—were anonymized after collection, and before the analysis, to comply with the sharing policies of the services.

Table 1 are provided the details of both datasets. We model them as *adoption logs*, L=(A,G,T), temporal ordered sets of triples of the form < *a*, *g*, *t* > where *a* ∈ *A* is an adopter, *g* ∈ *G* is the adopted item (the innovation) and *t* is the adoption time (i.e. <Jon, ACDC, 6/1/2017>).

**Table 1:** Datasets’ details. We report the time units used to build adoption trends: raw adoption log data of both datasets have a more fine grained time-scales as specified in dataset description.

Dataset	Items	Adopters	Adoptions	Timespan	AT Time unit
Last.fm	1 806	70 837	882 845	2 years	1 month
Coop	5 605	620 026	11 204 984	1 year	1 week

### 2.2 Defining success

Success is a concept not easy to quantify: indeed, several factors both exogenous (i.e., artist’s prizes or social impact) and endogenous (i.e., purchases’ volumes) can be used to measure it. To identify and characterize *Hit-Savvy*, we compare a few definitions of success: to guarantee context independency and objectiveness of our analysis we will focus only on endogenous ones. Indeed, the most straightforward way to measure the success is by looking at *volumes*: the number of listeners of an artist, as well as the number of purchases of an item, can be seen as natural proxies to evaluate and compare the attention received by different items of the same type. However, the volume is not the only way to capture success: another essential information is given by how adoptions spread over time. In order to identify a success measure alternative to volume, we analyzed the available data and devise a data-driven methodology that separate trend-successful items from trend-unsuccessful ones. The *adoption trend (AT)* of an *item* describes how its adoptions unfold through time: we can define it as,

**Definition 1 (Adoption Trend)** *Given an item g its adoption trend τ is a time series in which τ(t) identifies the percentage x of the total adoptions of g occurred at time t*.

As an example, consider an artist *A* and all its listeners during the observed period: the adoption trend of *A* describes, for each month, the percentage of listeners that listened *A* for the first time.

Indeed, the time unit chosen to define the adoption trend is context dependent. It is important to notice that reducing the temporal granularity of a given adoption trend (i.e., moving from a monthly scale to a weekly one) its overall shape will remain the same, only its smoothness will be affected. In our scenarios, to reduce noisy fluctuations while modeling adoption trends, we impose a weekly unit for Coop and a monthly one for Last.fm.

**Fig 1:**
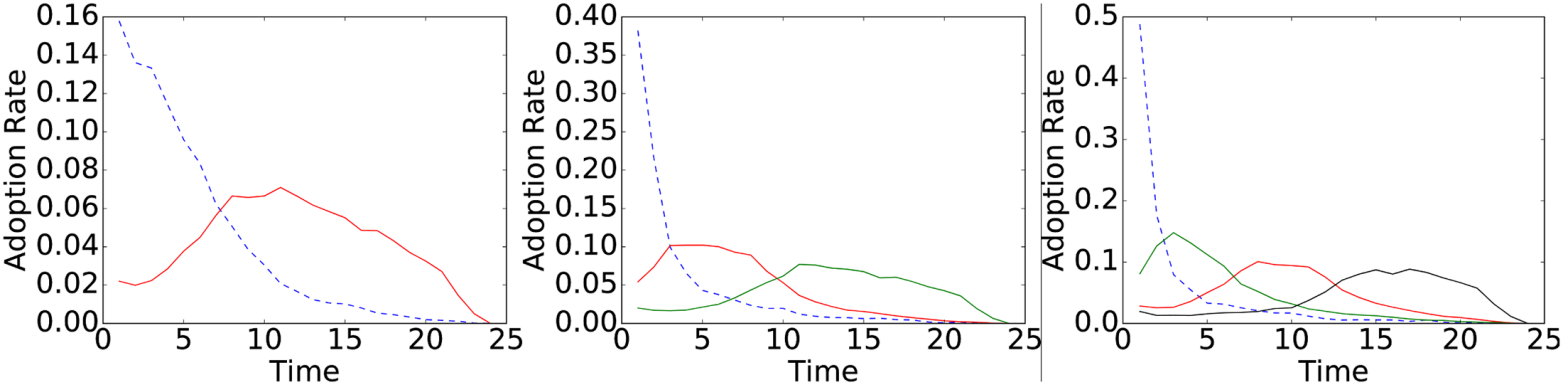
Last.fm trends analysis. Cluster medoids identified by k-means with DTW for *K* in [2, 3, 4]. SSE analysis identifies *k* = 2 as the best choice. The trends identified for *k* = 3, 4 do not unveil novel trend shapes, but only specialize the ones identified for *k* = 2.

Starting from each adoption log, we extracted items’ adoption trends and profiled them to identify recurrent patterns. To do so, we used k-means clustering with Dynamic Time Warping (DTW [7, 8]) as the distance function. We executed k-means using as a feature vector for each item *g* its observed trend *τ*. The study of cluster quality through SSE (Sum of Squared Errors) identifies, on both datasets, *k* = 2 as the optimal k-means parameter value. The medoids (i.e., cluster profiles) of the two well-separated clusters describe peculiar shapes: one expressing a sudden drop of the adoption rate, the other capturing an expanding trend. Fig 1 shows the medoids obtained on Last.fm, Coop ones behaving alike. In the same figure, we also report the Last.fm medoids for *k* = 3 and *k* = 4. In the latter scenarios, the additional profiles identify specializations of one of the medoids obtained for *k* = 2: in particular, they capture growing trends whose peak is shifted w.r.t. the one observed in the optimal solution and, most importantly, they do not describe novel previously unseen trend shapes. Several different clustering strategies can be employed to identify adoption trends families: we employed k-means with DTW since such methodology is widely used for clustering time series data. Indeed, we also tested a variant of k-means, namely k-shape [9], explicitly designed to group together similarly shaped time series. The results we obtained with such approach were indeed comparable to the k-means (both in the optimal number of clusters as well as shapes identified) ones, however, since k-shape implements a distance function that does not allineate shifted time series we choose to maintain the former approach as default clustering strategy.

**Fig 2:**
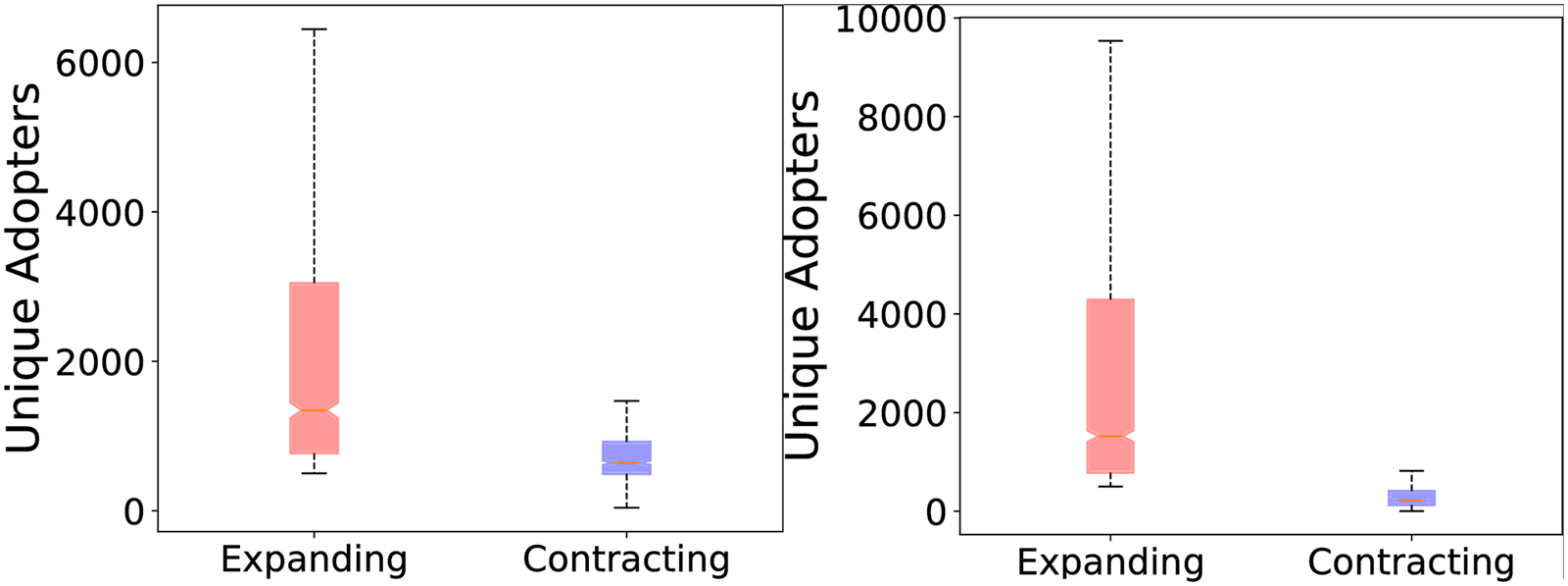
trends analysis. Comparison of volumes of expanding and contracting trends on Last.fm dataset (a) and Coop (b).

Since by construction the areas under trend curves are all equals to 1, we cannot compare the adopters’ volumes directly starting from items’ adoption trends. To understand if there exists a correlation between the shape of the adoption trend of one item and its adoption volume, we analyzed how volumes distribute w.r.t. the two clusters. The results, reported in Fig 2, show a clear tendency: items having expanding trends tend, on average, to have a broader diffusion than the others.

Notice that, to avoid biases while computing trend clusters as well as the need of temporal normalization, in this phase we employed only those items that were continuously adopted for at least half of the observed period (i.e., 1 year for Last.fm, 6 months for Coop). Thus, our data-driven definition of success discriminate successful and unsuccessful items by the shape of their trends:

**Definition 2 (Successful trends)** *Successful trends are the ones describing an increase of adoption rate through time capturing an expansion of individual items’ adopter base: e.g., all trends whose second derivative has negative value*.

**Definition 3 (Unsuccessful trends)** *Unsuccessful trends are the ones in which the adoption rate do not increase considerably over time or even reach an early maximum only to start to decrease rapidly: e.g., all trends whose second derivative has positive value*.

Following the previous example, an artist *A* will be considered successful if his adoption trend is concave, meaning that, as time goes by a growing number of individuals decided to listen to his music. Conversely, if *A*’s diffusion trend follows a convex shape, he will be considered unsuccessful since most of his adoptions happened early and he was unable to attract novel listeners as time goes by.

Independently from the success definition used, in the following, we will call *Hits* the successful items and *Flops* the unsuccessful ones. Moreover, we identify with *H* ⊆ *G* the set of *Hits* and with *F* ⊆ *G* the set of *Flops*.

Indeed, an entirely data-driven classification of items based on their adoption trends has an evident drawback: by definition, it cannot be considered *universal* nor *transferable* across different contexts. The proposed methodology can be applied to every kind of adoption log: however, it is possible that different data will generate clusters whose medoids profoundly differs from the patterns we identified in our analysis (both in number and shapes). In such scenarios we fall in an *“out-of-sample”* configuration w.r.t. the ones we encountered in both Last.fm and Coop: to move forward with the predictive model definition it will be thus necessary to label the identified clusters identifying a characteristic able to discriminate the item within them.

### 2.3 Identify *Hit-Savvy*

Once fixed the success definitions we can start searching for *Hit-Savvy*. To do so, we need to: (i) identify those adopters that adopt before the others successful items (the innovators) and (ii) measure their propensity to repeat such behavior in time.

#### Innovators

The classic approach used when addressing the problem of segmenting the customers of an item w.r.t. the time of their adoption lies in leveraging the definitions given by Rogers [4]. Rogers’ segmentation, which assumes a normal distribution shape for a generic adoption trend, defines the *innovators* as the first 2.5% of the item adopters. Since our data-driven analysis revealed the absence of such peculiar distribution of adoptions we decided to employ an alternative more conservative method to identify such interesting segment of the population. We thus define:

**Definition 4 (Innovators)** *Given an adoption trend τ of an item g we consider innovators all those adopters that adopt g strictly before the global maxima of τ*.

The proposed definition does not impose a fixed threshold and applies to all the different trends that can be observed in real data. Indeed, this criterion potentially generates a wider set of innovators than the Rogers’ one: for these reasons, to support our alternative definition, in Section 4.1 we will compare it with Roger’s while predicting successful items.

#### Hit-Savvy

Once identified the *innovators* we need to quantify their intrinsic individual propensity to adopt *Hits*. To do so we define *HF-propensity*:

**Definition 5 (HF-Propensity)** *Given an adopter a and (i) f the number of* Flops *he adopted, (ii) h the number of the* Hits *he adopted as* innovator *and (iii) k the number of* Hits *he adopted after they reach their global maxima, the* HF-propensity *of a is*:

$$\begin{array}{c} HF\left(a\right)=\frac{h-k-f}{h+k+f} \end{array}$$ (1)

*HF lies in* [−1, 1]: *its value is maximised when a adopts only* Hits *as innovator, minimised when his adoptions regard prevalently* Flops *as well as* Hits *but as latecomer*.

Once fixed a way to measure the propensity toward the exclusive adoption of successful items we can formally define the *Hit-Savvy*:

**Definition 6** (Hit-Savvy) *Given an adoption log* L=(A,G,T), *a set of successful items H* ⊂ *G we define* Hit-Savvy *all those adopters a* ∈ *A for which HF* > 0.

The farther the HF-propensity value is from 0, the stronger the *Hit-Savvy* strength: we identify with *HT* the set of *Hit-Savvy* and with *FT* the set of *Flop*-adopters (e.g. adopters having *HF* < 0). Adopters having *HF* = 0 are considered *neutral* signals since they do not show any special propensity toward neither *Hits* nor *Flops*. In the following analysis, we will discard neutral adopters since they cannot be considered discriminatory indicators.

Indeed, since the *HF* measure is not weighted on the number of adoptions, an adopter that has adopted a single successful (unsuccessful) items will receive the highest (lowest) value in the range. Even though this choice appears to be counterintuitive, such scenario represents a rare event in the data we analyzed and, most importantly, it does not affect the semantics we search for while defining our *Hit-Savvy*. An adopter must be considered a *Hit-Savvy* even if he only adopted a single item among thousands available if, later on, such item reached success. Indeed, it is likely that such adopter will not be able to contribute to identifying successful items in the future due to his reduced adoption activity. However, his behavior is similar to all other *Hit-Savvy*. As previously discussed *Hit-Savvy*, conversely from Tetlock’s super-forecasters, are not driven by the desire to identify successful items: they only chose to adopt something that they like or need.

### 2.4 *Hit-Savvy*: Who are they?

**Fig 3:**
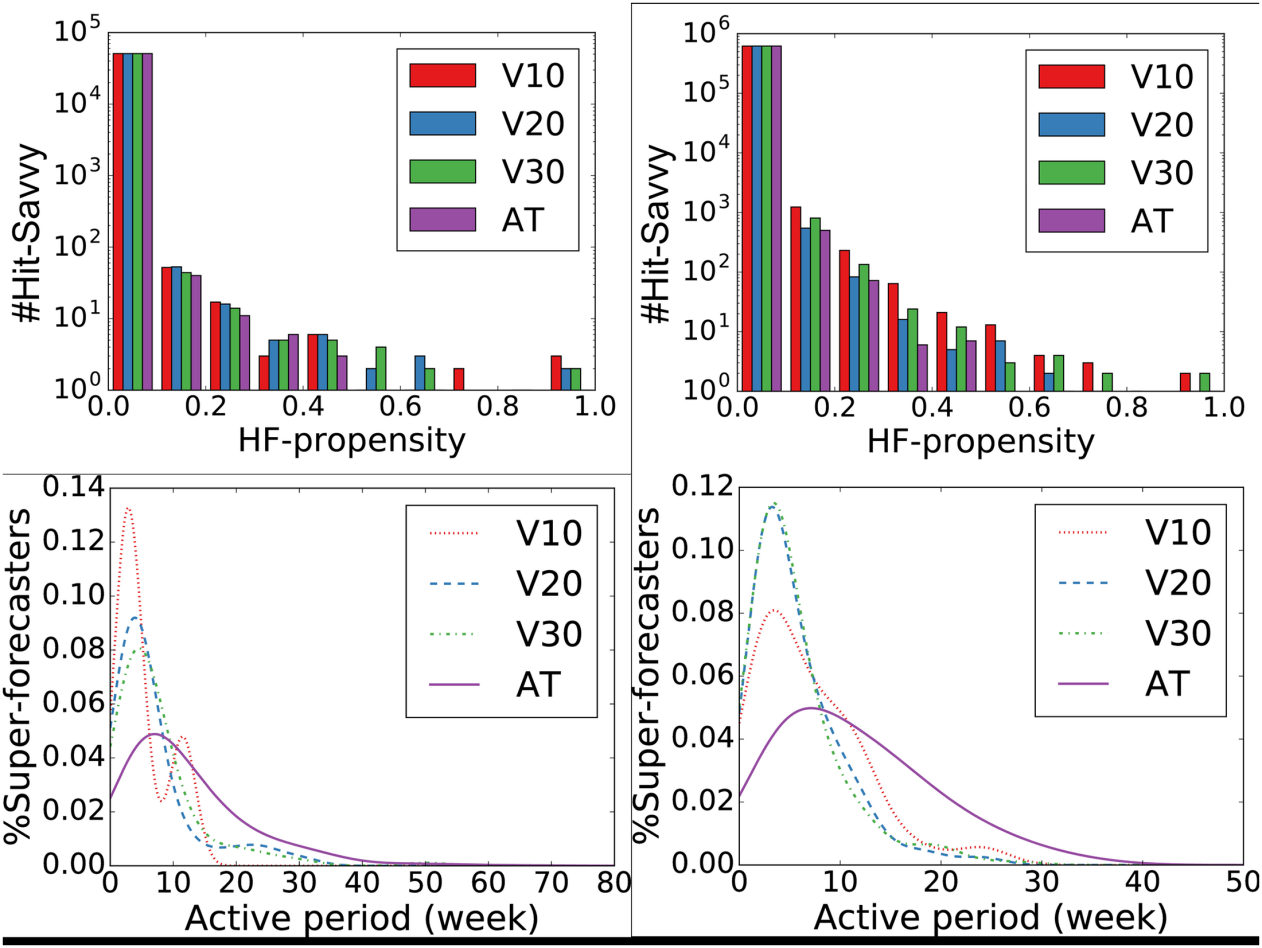
*Hit-Savvy*: HF-propensity and active periods. *Hit-Savvy* vs. HF-propensity in Last.fm (top left) and Coop (top right). Distribution of *Hit-Savvy*’ active periods length for Last.fm (bottom left) and Coop (bottom right).

Once fixed both the success definitions and the criterion to identify *Hit-Savvy* several questions arise: (i) do *Hit-Savvy* exist? (ii) how many of them are we able to identify? To better highlight the impact different success definitions have on *Hit-Savvy* we compare four of them: three based on volume (V10, V20, V30) that define as Hits respectively the top-10%, top-20%, and top-30% most adopted items, and AT, the data-driven trend-based we proposed. We can observe in Table 2 that, disregarding the dataset and the success definition used, HF-propensity often identifies as *Hit-Savvy* less than 1% of the total adopters: a niche of individuals still able to adopt a high percentage of *Hits*. In particular, the induced coverages fluctuate across the 12-42% on Last.fm and the 4-9% on Coop when success is defined as a function of volume, while they reach respectively the 22% and 79% if success is modeled using AT. In Fig 3 (top) we show the binned distributions of the HF-propensity for the identified *Hit-Savvy* while varying the success definition. For both Last.fm and Coop, a neat peak for low HF-propensity scores emerges (in the range (0, 0.1]), implying that it is very rare to identify within *Hit-Savvy* adopters that adopt Hits exclusively. Moreover, all the analyzed success definitions induce similar right skewed long tail shaped distributions for such indicator, thus not excluding the existence of few, precious, very strong *Hit-Savvy*.

**Table 2:** *Hit-Savvy* statistics. For each dataset and success definition are reported: the number of *Hit-Savvy* |*HT*|, the number of successful items |*H*| and the coverage induced on them by *Hit-Savvy*. Within bracket are reported their percentage over all the adopters and items respectively.

Dataset	Def.	|*HT*|	|*H*|	Coverage
Last.fm	V10	9 (0.01%)	181 (10%)	12.77%
Last.fm	V20	48 (0.06%)	361 (20%)	28.53%
Last.fm	V30	96 (0.13%)	541 (30%)	42.69%
Last.fm	AT	1 034 (1.45%)	1 000 (55%)	78.67%
Coop	V10	28 (0.004%)	560 (10%)	4.28%
Coop	V20	79 (0.013%)	1 121 (20%)	6.42%
Coop	V30	207 (0.033%)	1 681 (30%)	9.39%
Coop	AT	54670 (8.817%)	1 639 (29%)	21.55%

Indeed, our experiments suggest that *Hit-Savvy* exist. To understand if they can be fruitfully used to forecast success, we still need to answer an important question: for how long a generic *Hit-Savvy* adopt as such? So far we computed the HF-propensity for each adopter over his complete history of adoptions: to understand if a *Hit-Savvy* adopts as such only few random times across his history or stably performs prevalently Hit adoptions as an innovator, we computed his HF-propensity on a weekly basis. Let $HF\left(a\right)=[HF_{t_{0}}\left(a\right),\ldots ,HF_{t_{n}}\left(a\right)]$ be the ordered list of weekly HF-propensity of the adopter *a* ∈ *A* and [*t*_0_, …, *t*_*n*_] the weeks in which *a* makes at least an adoption, we define as *Hit-Savvy*’s active period the average number of consecutive weeks in HF(a) having HF-propensity greater than 0. In Fig 3 (bottom) we show the probability distribution of *Hit-Savvy*’ active periods computed on both datasets varying the success definitions. Two clear patterns emerge: (i) as happened for the HF-propensity score we observe peaked long-tailed distributions (having their modes within 5-10 weeks in Last.fm and 5-6 weeks on Coop) and, (ii) AT is able to guarantee the identification of *Hit-Savvy* with the longest active periods (∼80 and ∼45 weeks on, respectively Last.fm and Coop).

## 3 Predicting success

Certainly, the road to success is not an easy one. To decide, by observing only a short time-bounded adoption log, if a novel item is destined to become a *Hit* (or be doomed to be just another of many *Flops*) we need to extract meaningful information from known examples and to design a reliable forecasting model. Music companies, as well as online music providers (Spotify, Apple Music…), can greatly benefit from being able to discriminate *Hit* artists from *Flop* ones during the early stages of their distribution so to design ad-hoc marketing and advertising strategies. *Hit-Savvy* play a huge role in this process: being able to identify a sufficiently broad and stable group of individuals capable of spotting success before others is one of the dreams of every marketing department. Indeed, we have seen that different definitions of success generate different sets of *Hit-Savvy* that vary both in volume and stability. Volume-based definitions, even if immediate and of natural formulation, have an unpleasant drawback: the reduced set of positive, successful items they define causes the identification of very few and volatile *Hit-Savvy*. Such reduced set of special users, although being very strong signal for success, are usually not enough to make predictions: for this reason in the following, we will adopt our data-driven trend-based definition of success to formulate our predictive model. The approach we propose, *Hits&Flops* (code available at https://goo.gl/f36Unv), follows a simple rationale: identify the smallest set of *Hit-Savvy*, as well as *Flop*-adopters, that allow discriminating Hits from Flops and leverage them to make predictions. Our predictive model, namely *Hits&Flops*, can be applied independently from the chosen success definition.

### 3.1 Hits&Flops: Learning Hitters and Floppers

So far we have measured the *adopters* propensity toward *Hits* and *Flops* and thus transformed them into indicators. Our aim is now to use such indicators to forecast the success of novel, previously unseen, items. As we have seen in 2.4 HF-propensity distributions is right skewed in both datasets: since we aim to reduce the noise weak signals can generate while used to make prediction we target the minimum sets of *Hit-Savvy* as well as of *Flop*-adopters able to discriminate both *Hits* and *Flops*.

The task of identifying such subsets can be naturally formulated as a *Weighted Multi-Set Coverage* (WMSC) problem. To comply with WMSC, we build two bipartite graphs: one connecting each *Hit-Savvy* in *HT* to the successful items he adopted, and the other connecting each *Flop*-adopters in *FT* to his unsuccessful items. On each of such topologies, we search for the subset of adopters that guarantees a constrained minimum coverage of the connected items. A valid coverage needs to satisfy the WMSC formulation:

$$\begin{array}{cc} \text{min}{}_{x_{1}\cdots x_{\left|X\right|}} & \;\sum_{i}x_{i}\\ \text{subject}\;\text{to}\; & \;\forall \;i,j:x_{i}=e_{i,j}\\ & \;\forall \;j:\sum_{i}e_{i,j}\geq y_{j}\beta \\ & \;\sum_{j}y_{j}\geq \alpha \left|Y\right|\\ \text{where}\; & \;x_{i},e_{i,j},y_{j}\in \left\{0,1\right\},\;\alpha ,\beta \in \left(0,1\right] \end{array}$$

where *x*_*i*_, *y*_*j*_, *e*_*i*,*j*_ are binary variables modelling each potential *adopter* (belonging to the *adopter* set X), item adopted (belonging to the item set Y), and edge of the bipartite graph. The first constraint ensures that a selected *adopter* contributes to the coverage of each of the item he adopts. The second constraint ensures that selected *adopters* cover at least *β*% of the sum of all incoming edges to each item. The third constraint ensures that the selected *adopters* collectively cover at least *α*% of the set of items.

We designed a greedy approach to solving WMSC (Algorithm 1) that selects a subset of adopters which satisfies the *α* and *β* thresholds. In order to easily manage adoption log structures, in the pseudocode we make use of the following notation: let *a* ∈ *A* be an adopter, *g* ∈ *G* an item, A_*_ ⊆ *A* and G_*_ ⊆ *G* two sets then, (i) *ω*(*a*, G_*_) identifies the set of items in G_*_ adopted by *a* and, (ii) *ψ*(*g*, A_*_) identifies the adopters of *g* within A_*_.

**Algorithm 1** WMSC greedy

**Require:** *B* = (*A*, G_*_): bipartite graph composed by adopters *A*, and items G_*_; *c* a sorting strategy based on degree and *HF*-propensity; *α* global coverage threshold; *β* local coverage threshold.

1: Indicators = [ ], covered_items = [ ]

2: *A*_*s*_ = sort(A, c)

3: **for** a ∈ *A*_*s*_ **do**

4:  **if** |covered_items| < *α* |G_*_| **then**

5:   *G*_*a*_ = *ω*(*a*, G_*_)

6:   **for** g ∈ *G*_*a*_ **do**

7:    **if** |*ψ*(*g*, *Indicators*)| < *β* degree(g) **then**

8:     **if** a ∉ Indicators **then**

9:      Indicators.add(a)

10:    **else if** g ∉ covered_items **then**

11:     covered_items(g)

12:  **else**

13:   **return** Indicators

14: **return** [ ]

**Fig 4:**
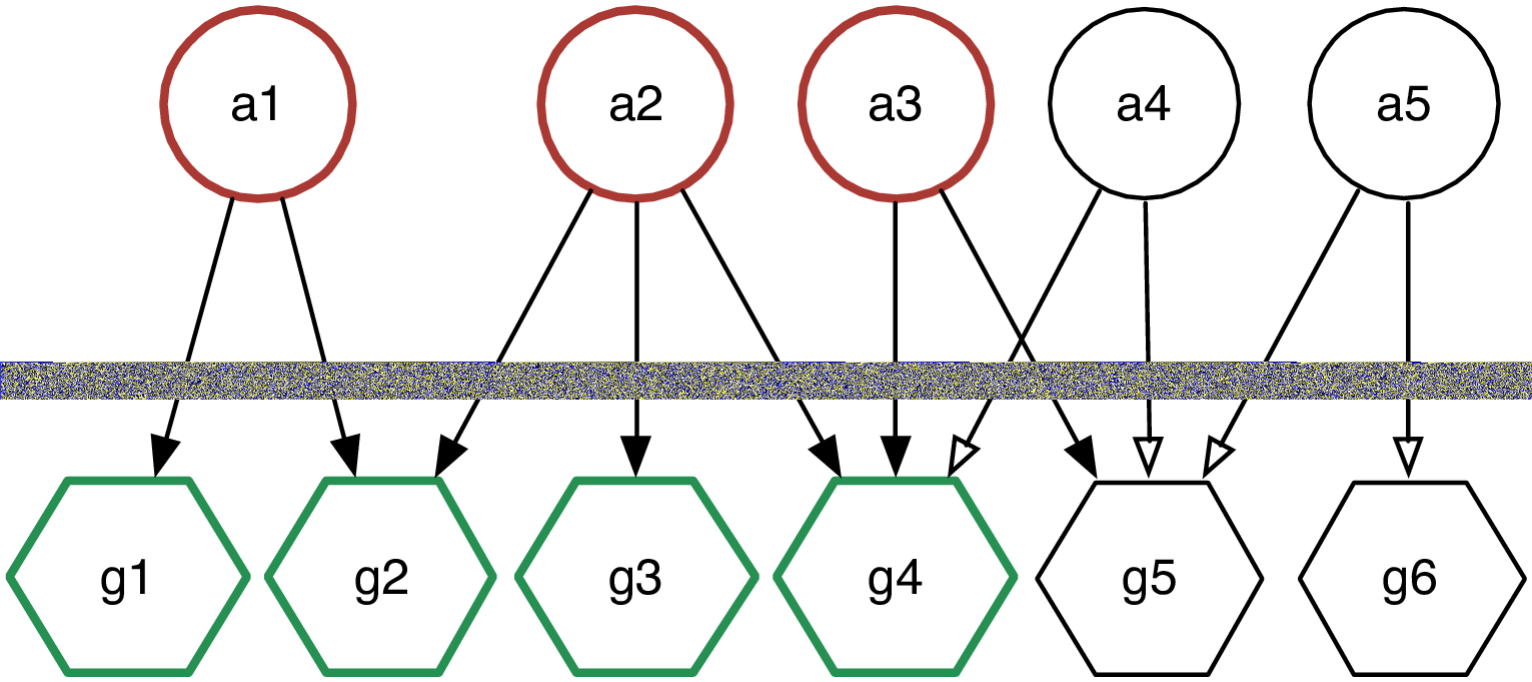
WMSC toy example. WMSC with *α* = *β* = 0.6: in red the selected adopters, in green the covered items.

Our greedy approach takes care to sort the adopters to be selected for the covering: it selects the adopters maximizing their degree and *HF-propensity* to cover *Hits* and maximizing their degree while minimizing their *HF-propensity* to cover *Flops*. Doing so, we impose the identification of a local maximum composed by highly discriminative *Hit-Savvy* (*Flop*-adopters). In Fig 4 is shown the WMSC coverage for a simple bipartite graph: in the toy example, to simplify the analysis, the *HF-propensity* is considered equal for all the adopters (e.g. only a degree ordering is exploited in the selection phase). Since the *α* and *β* parameters deeply affect the coverage produced by WMSC, to identify the optimal *HT* and *FT* subsets we instantiate multiple runs of WMSC applying a grid search strategy. Such strategy results in two sets of solutions: (i) one for *Hit*-coverages, $HT=\{HT_{0},\ldots ,HT_{n}\}$ with *HT*_0_, …, *HT*_*n*_ ⊆ *HT* and, (ii) the other for *Flop*-coverages $FT=\{FT_{0},\ldots ,FT_{n}\}$ with *FT*_0_, …, *FT*_*n*_ ⊆ *FT*. Given a valid solution $HT_{i}\in HT$, let *H*_*i*_ ⊆ *H* be a the subset of items covered by adopters in *HT*_*i*_. Symmetrically, given a valid solution $FT_{j}\subseteq FT$, let *F*_*j*_ ⊆ *F* be a the subset of items covered by adopters in *FT*_*j*_. Among the solutions within HT and FT we select *HT*_*i*_ and *FT*_*j*_ that maximise |*H*_*i*_| and |*F*_*j*_| while having minimal cardinality: we call them $\hat{HT}$ and $\hat{FT}$. Such selection procedure acts as a feedback control loop that implicitly supports the identification the optimal values of *α* and *β* among the ones tested by the grid search strategy.

Finally, we call *Hitters* all the filtered and reliable *Hit-Savvy* within $\hat{HT}$. Symmetrically, we call *Floppers* all the *Flop*-adopters within $\hat{FT}$.

### 3.2 Hits&Flops: Forecast model

Once learned the Hitters and Floppers to predict whether a novel item will be either a *Hit* or a *Flop* we proceed as follows.

For each successful item *g* ∈ *H* let *π*(*g*) be the number of its adopters in $\hat{HT}$ and Π the probability distribution of *π*(*g*) across all the items in *H*. Symmetrically, for each unsuccessful item *g* ∈ *F* let *δ*(*g*) be the number its adopters in $\hat{FT}$ and Δ the probability distribution of *δ*(*g*) across all the items in *F*. We call $\hat{m}$ ($\hat{n}$) a user specified threshold over Π (Δ).

We define the *Hits&Flops* predictor as a two step procedure:

1. **Specialized classification.** Two distinct classifiers are built: one to forecast *Hits* (leveraging $\hat{HT}$ set and Π distribution), the other to forecast *Flops* (leveraging the $\hat{FT}$ set and Δ distribution).
2. **Synthesis.** A meta-classifier combines the predictions of specialized classifiers and generates the final prediction for each novel item.

Let g_*_ be a previously unseen item and A_*_ the set of its adopters during a limited time window starting from its first appearance: the rationale behind the specialized predictors is the following:

**Definition 7 (Hits-classifier)** $H_{p}$ *returns a positive class (Hit) iff* $\pi \left(g_{*}\right)>\hat{m}$ *where π*(g_*_) *is computed over* $A_{*}\cap \hat{HT}$.

**Definition 8 (Flops-classifier)** $F_{p}$, *returns a positive class (Flop) iff* $\delta \left(g_{*}\right)>\hat{n}$ *where δ*(g_*_) *is computed over* $A_{*}\cap \hat{FT}$.

Since those classifiers give only partial predictions their outcomes need to be combined by a meta-classifier:

**Definition 9 (Hits&Flops)** *Given the Hits and Flops predictors* $H_{p}$ *and* $F_{p}$, *we define the rule based meta-classifier as*:

1. *if* $H_{p}\left(g_{*}\right)\to Hit$ *and* Fp(g*)→Flop, g_*_ *is a future* Hit;
2. *if* Hp(g*)→Hit *and* $F_{p}\left(g_{*}\right)\to Flop$, g_*_ *is a future* Flop;
3. *if* $H_{p}\left(g_{*}\right)\to Hit$ *and* $F_{p}\left(g_{*}\right)\to Flop$:
  - *if* $\pi \left(g_{*}\right)-\hat{m}>\delta \left(g_{*}\right)-\hat{n}$, g_*_ *is a future* Hit;
  - *if* $\pi \left(g_{*}\right)-\hat{m}=\delta \left(g_{*}\right)-\hat{n}$, *the observed adoptions are still not enough to make a forecast*;
  - otherwise g_*_ *is a future* Flop;
4. *otherwise, the observed adoptions are still not enough to make a forecast*.

**Fig 5:**
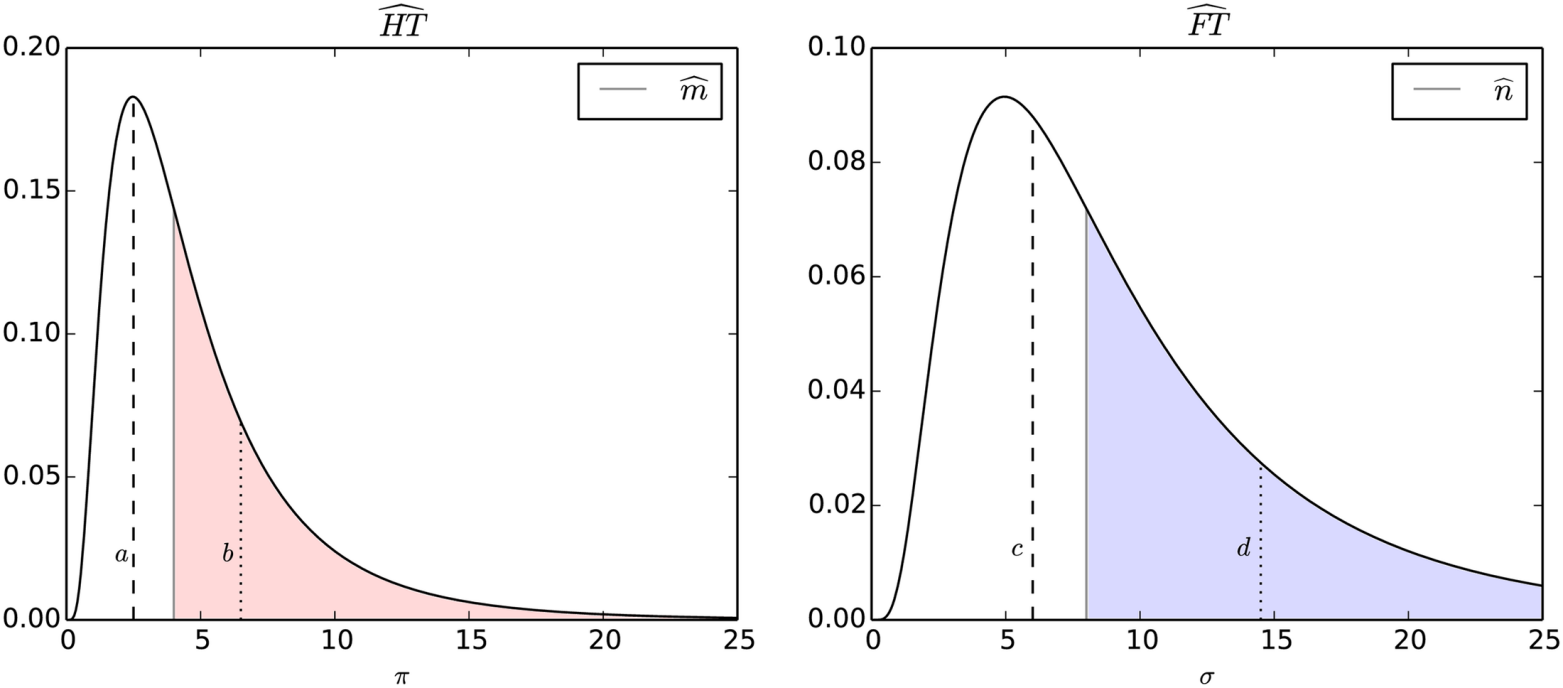
Probability distributions Π and Δ. The thresholds are identified with $\hat{m}$ and $\hat{n}$ respectively.

To exemplify the rules of the meta-classifier in Fig 5 are depicted the distributions Π and Δ on Last.fm. The first (second) rule identifies an item g_*_ having *π*(g_*_) = *b* (*π*(g_*_) = *a*) at the right (left) of $\hat{m}$, and *δ*(g_*_) = *c* (*δ*(g_*_) = *d*) at the left (right) of $\hat{n}$. In these cases, the *meta-classifier* returns the positive class identified by the corresponding classifier. The third rule models the situation in which both the classifiers return a positive class for the item g_*_ (in the example, *π*(g_*_) = *b* and *δ*(g_*_) = *d*). In this case, the *meta-classifier* will return the class identified by the greater distance between the thresholds and the corresponding value of *π*(g_*_) or *δ*(g_*_) (in the specific case will classify g_*_ as *Flop*, since $\left(b-\hat{m}\right)<\left(d-\hat{n}\right)$), and in case the two distances are equivalent, it will not return a classification. Finally, the fourth rule models the case in which both the classifiers do not return a positive guess (in the example, *π*(g_*_) = *a* and *δ*(g_*_) = *c*). In this case, a classification is not provided: since we are dealing with *partial* observations we choose not to classify the instances for which we do not have enough data.

We designed a meta-classifier, avoiding the use of classic approaches (e.g., Decision Trees) since the peculiar formulation of the problem allows, in the absence of sufficient observations, to suspend the class prediction (generating a “third”, temporary, class that is not described by any example in the training set). In the following, we adopted as $\hat{m}$ and $\hat{n}$ the median values of the two distributions after a stage of parameters tuning that highlighted their ability to guarantee highest predictive performances on training and test sets of the analyzed datasets. Indeed, being a data-driven analysis, different datasets could require different thresholds values to fine-tune the classification process. For such reason, in our framework, we leave $\hat{m}$ and $\hat{n}$ as model parameters, allowing the analyst to fit them to the distributions observed in the data as well as to specific analytical needs.

## 4 Evaluation

In 4.1 we apply *Hits&Flops* on both Last.fm and Coop datasets and compare its predictions with the ones produced by its competitors. In 4.2, we statistically validate the significance of our results. Finally, in 4.3 an in-depth analysis of the Last.fm case study is provided.

### 4.1 Experimental results

We tested *Hits&Flops* against two different classes of competitors: (i) time-series forecasting and (ii) innovator based supervised learning predictors. A classic time-series based strategy to address our problem is to (i) reconstruct (forecast) a complete trend moving from its partial observation, (ii) estimate its distance from the medoids of the previously identified clusters and (iii) assign it to the closest one, thus determining if the new item will be either a *Hit* or a *Flop*. To do so, we adopt as a first set of competitors two time-series forecasting techniques: one that takes into account seasonality while reconstructing the adoption trend, Fast Fourier Transform (*FFT*), and one that does not, Moving Average (*MA*).

*Hits&Flops* does not only generates predictions for novel items: it also identifies the sets of *Hitters* and *Floppers*, valuable adopters that can be monitored and used to influence marketing strategies. For this reason, we tested our predictive approach also against competitors able to produce such valuable byproduct. In particular, we compared to the following innovator-based supervised learning strategies:

1. *ER*: the prediction is made using only the innovators extracted with the 2.5% Rogers’ threshold. For this approach, an item will be defined a *Hit* iff at least one of its observed adopters is a known *Hit-Savvy*, a *Flop* otherwise;
2. *ER-H&F*: the initial *HT* set for *Hits&Flops* is identified by using the threshold proposed by Rogers (e.g., the first 2.5% of the item adopters);
3. *HA-H&F*: a variant of *Hits&Flops* in which the coverage on the bipartite graph is obtained not through the proposed WMSC heuristic but by selecting the top-k scorers of Hubs&Authorities [10](where the optimal k is identified using the same procedure to optimize *α* and *β*);

To evaluate the effectiveness of the aforementioned strategies we design our experiments temporally dividing each dataset into a training set (items that appear for the first time within the first 60% of the dataset observation period) and test set (items appearing for the first time in the residual 40%). We considered as partial observation windows for the items of the test set respectively: 2 months in Last.fm and 4 weeks in Coop.

#### Hits&Flops

First of all, we need to justify the rationale behind the proposed approach: why not to use directly HF-propensity scores to make predictions? To answer such question, we built a simple classifier that exploits directly the indicators, as computed in 2.3, by combining them linearly. In such scenario, a novel item will be labeled as *Hit* if the sum of the HF-propensities of its observed adopters is greater than 0, *Flop* otherwise. Unfortunately, such approach is not able to make predictions for *Hits* due to the highly unbalance among the computed indicators (*Hit-Savvy* are always less than 1%). The introduction of the WMSC step is a way to address such unbalance: are then $\hat{HT}$ and $\hat{FT}$ sufficient to make predictions without leveraging the meta-classifier? We use the HF-propensity scores for adopters in such sets to compute the same linear combinations: even in this case we get very low Recall on the *Hit* class: 0.023 on Last.fm and 0.061 on Coop. Such low performances are due to the right skewness of the HF-propensity distribution for Floppers which tends, conversely from Hitters, to be polarized toward extreme values.

#### Comparative analysis

Table 3 report the performances of the proposed approach as well as of its competitors on the two datasets. Time series forecasting approaches, either susceptible to seasonality (*FFT*) or not (*MA*), are constantly outperformed by *Hits&Flops*. Moreover, we can observe two distinct, but related phenomena: (i) *FFT* always shows higher reliability while predicting *Hits* (higher PPV and Recall) w.r.t. *MA* and (ii) *MA* always outperforms *FFT* while predicting *Flops* (higher NPV and Specificity). Such results are consistent with the patterns expressed by the trends belonging each cluster: *Flops* are not subject to seasonality since their trends experience a contraction.*Hits*, conversely, are characterized by manifold trends that can have multiple local minima and maxima.

**Table 3:** PPV, NPV, Recall and Specificity comparison for the analyzed forecasting approaches.

**A.** *PPV*—Positive Predictive Value, calculated as $\frac{TP}{TP+FP}$								
**Dataset**	***H&F***	***ER*** ***H&F***	***HA*** ***H&F***	***ER***	***MA***	***FFT***	**NM**	
							*avg*	*std*
**Last.fm**	.766	.290	.685	.000	.026	.327	.644	.002
**Coop**	.781	.825	.959	.000	.002	.247	.547	.010
**B.** *NPV*—Negative Predictive Value, calculated as $\frac{TN}{TN+FN}$								
**Dataset**	***H&F***	***ER*** ***H&F***	***HA*** ***H&F***	***ER***	***MA***	***FFT***	**NM**	
							*avg*	*std*
**Last.fm**	.471	.392	.309	.351	.269	.197	.026	.046
**Coop**	.316	.384	.308	.292	.106	.004	.051	.028
**C.** *Recall*—True Positive Rate, calculated as $\frac{TP}{TP+FN}$								
**Dataset**	***H&F***	***ER*** ***H&F***	***HA*** ***H&F***	***ER***	***MA***	***FFT***	**NM**	
							*avg*	*std*
**Last.fm**	.520	.006	.148	.000	.004	.153	.990	.019
**Coop**	.586	.031	.081	.000	.001	.135	.818	.043
**D.** *Specificity*—True Negative Rate, calculated as $\frac{TN}{FP+TN}$								
**Dataset**	***H&F***	***ER*** ***H&F***	***HA*** ***H&F***	***ER***	***MA***	***FFT***	**NM**	
							*avg*	*std*
**Last.fm**	.727	.970	.832	1.00	.701	.397	.007	.015
**Coop**	.522	.983	.990	1.00	.286	.008	.361	.024

Focusing our attention on the innovators-based methods, we observe that the *ER* approach does not obtain any valuable results w.r.t. the precision of the predictions (i.e., low scores of PPV and NPV). Due to the non-gaussian adoption distribution of the analyzed datasets, the choice of identifying the *innovators* with a fixed 2.5% threshold and to use them—without further refinement—to forecast success leads to overestimating the *Flops* (as indicated by the values of Recall and Specificity). To understand if the innovators identified by *ER* are still valuable, we build upon them a modified version of our model, namely *ER-H&F*. Since we aim to achieve high predictive precision especially in the prediction of successful items (PPV score) and guarantee that we can correctly identify a significative percentage of them, we can state that our method is always capable of outperforming such competitor. The limitation of *ER-H&F*, inherited from *ER*, is due to the choice of identifying innovators using a fixed threshold. However, *ER-H&F* outperforms *ER*. Such result confirms that not all innovators are good indicators of success and, as shown by the experimental results, our methodology is capable of recognizing and exploiting the ones which can be reliably used as signals to make predictions. Moreover, considering the results provided by *HA-H&F* we observe how this variant is able to reach high PPV and Specificity at the cost of very low NPV and Recall. This latter result highlights that the proposed WMSC greedy solution which, conversely from Hubs&Authorities, takes into account *HF-propensity* plays an important role in the definition of the Flop classifier. In conclusion, *Hits&Flops* is always able to outperform the competitors producing effective predictions. Moreover, in the performed experiments our meta-classifier is able to assign a class to, respectively, 97% new artists in Last.fm and 94.4% new Coop products (among the ones covered by the *Hit-Savvy* and identified in Section 2.4).

### 4.2 Null model analysis

The main hypothesis behind *Hits&Flops* is that there are very special adopters, the *Hit-Savvy*, which tend to choose and adopt prevalently successful items. To validate that the results obtained from our case studies are statistically significant, we need to prove that they are driven not by chance but, conversely, by adopters’ explicit behaviors. To do so, we perturbed our datasets, executed *Hits&Flops* on the outcome of such process and statistically tested the results obtained against the ones we obtain on real data. The randomisation we applied targeted adopters choices while preserving two important characteristics of the original data: (i) the adoption trend distribution for each item (e.g., *Flops* and *Hits* remained as such); (ii) the original number of adoptions and relative temporal distribution for each adopter.

The performed transformation acts as our Null Model (its pseudocode is reported in Algorithm 2): it ensures that both adopters and items maintain their characteristic distributions, by destroying only the temporal sequence of adoptions and individuals choice on the item to adopt. The final adoption log describes adopters that choose items without any particular personal preference. In detail, Algorithm 2 takes as inputs two maps U and V which relates, respectively, each adopter and each pair < *item*, *time* > to their number of adoptions. Such procedure builds a matrix of size |U|×|V| and populates it by randomly selecting indices from the two maps, taking care of maintaining the defined constraints.

**Algorithm 2** Null Model Generator

**Require:** U: map of *adopter* → *number of items*; V: map of <*item*, *time* > → *number* *of* *adoptions*.

1: m = matrix(|U|, |V|)

2: **while** |U| > 0 and |V| > 0 **do**

3:  a = random(U.key)

4:  < *g*, *t* > = random(V.key)

5:  m[a][< *g*, *t* >] += 1

6:  U[a] -= 1, V[< *g*, *t* >] -= 1

7:  **if** U[a] == 0 **then**

8:   U.remove(a)

9:  **if** V[< *g*, *t* >] == 0 **then**

10:   V.remove(< *g*, *t* >)

11: model = []

12: **for** (a, < *g*, *t* >) ∈ m **do**

13:  **if** model[a][< *g*, *t* >] > 0 **then**

14:   model.append(a, g, t)

15: **return** model

#### Statistical significance

We generated 10 different null models for each dataset which were used to instantiate *Hits&Flops* following a 10-fold cross-validation strategy, thus testing it over 100 different combinations of training-test sets. In Table 3 with *NM* are identified the average performances of our approach on the transformed data, along with their standard deviations. Computing the z-test to compare the performances achieved by *Hits&Flops* on real data and null model generated ones we obtain the values shown in Table 4. Given the low standard deviation on the null models, the z-scores obtained reach values that are remarkably far from the ones given by *Hits&Flops*: we can thus conclude that the results obtained by *Hits&Flops* on real data are statistically significant. As an example, the value 61.0 for PPV (Positive Precision) in Last.fm dataset in Table 4 means that *Hits&Flops*’ Precision is 61.0 Null Model’s Standard Deviations far from the Null Model Precision’ average ($61.0=\frac{.766-.644}{.002}$). We then reject the null hypothesis that the observed performances can be explained by the null model.

**Table 4:** *H&F* z-test. The lowest z-score obtained, 5.4, lead to a p-value (one-sided) of 3.332e-8: z-scores higher than 8.3 lead to p-value equals to 0.

Dataset	PPV	NPV	TPR	SPC
**Last.fm**	61.0	9.7	24.2	48.0
**Coop**	23.4	9.5	5.4	10.9

### 4.3 Case study: Last.fm

In order to better discuss the performances of *Hits&Flops* here we focus on its results on the Last.fm dataset. We analyze this particular scenario because Last.fm adoption logs are very dense and accurate and the concept of novel item (new artist) is crisp and not affected by biases such as the *brand-effect* in the retail market data. Indeed, in a retail market scenario, the success of a new product (i.e., a new type of *“pasta”*) is often strictly related to the overall success of its brand (i.e., *“Barilla”*). Due to such dependency, on average, it is not easy to separate real novel Hits from those products whose success is due to the users’ attachment to a brand.

**Fig 6:**
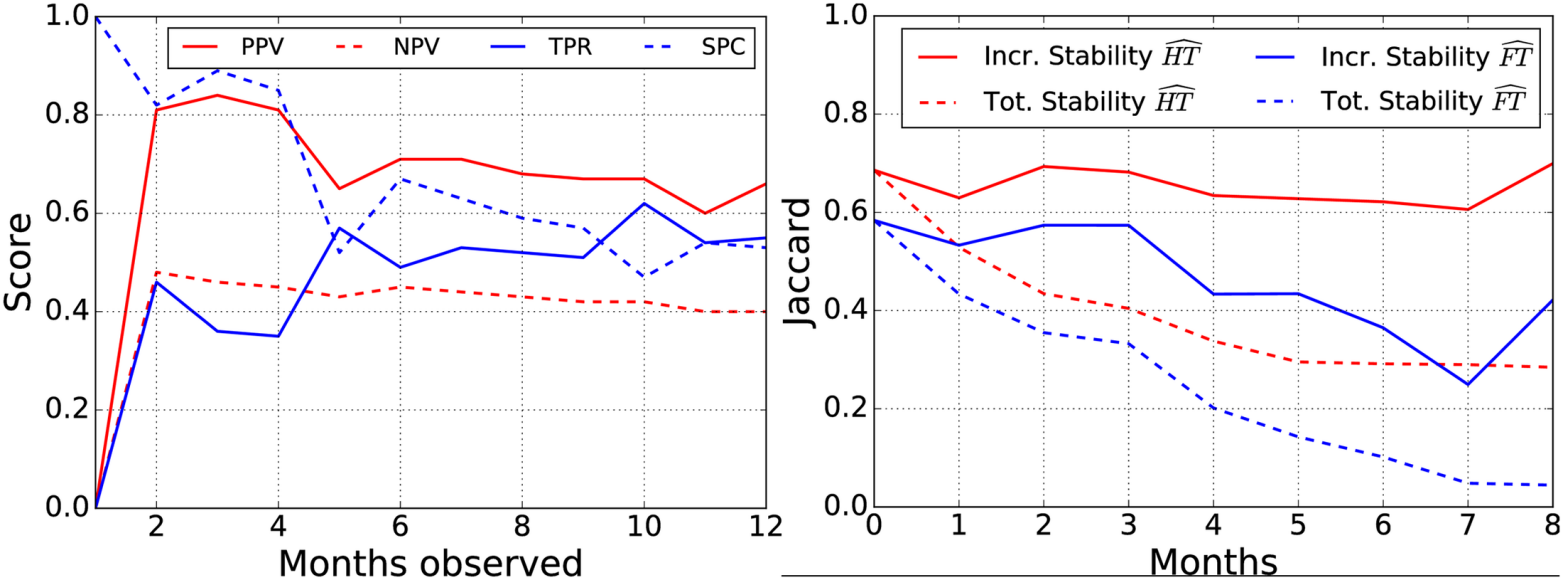
Predictive accuracy stability. Predictive accuracy varying the observation period for the Last.fm dataset (left). Stability of Hitters and Floppers sets across time: the analyzed models were learned with a 9-15 split using a sliding window (right).

One of the main questions that arise when forecasting novel artists’ success is: *for how long do I need to observe his listeners to make a reliable prediction?* To study the effect that time has on the predictive performances of *Hits&Flops*, we run it on Last.fm varying the observation period for novel artists. We report in Fig 6 (left) the trends of PPV, NPV, Recall, and Specificity obtained applying our model with increasingly longer observation periods (ranging from 1 month to 1 year). We notice that the main effect of a protracted observation is to introduce variability on Recall: with more observations, we are likely to identify a higher percentage of *Hits* preserving a high PPV and NPV. Conversely, Specificity stabilizes on the long run. Hence, for this specific case study, the optimal observation window can be reasonably fixed at 2 months since, with such settings, we observe the best predictive power. Such results are due to the fact that rely on few observations lowers the probability of encounter Hitters adoptions (and thus identify *Hits*) while, on the other hand, excessively extend the observation window is likely to induce over-representations of *Floppers*.

Next, we split our data using an incremental temporal window to represent the past—while maintaining the identified optimal observation window length. We learn our model on those artists (and related listening) that appear during the first 3-6-9-12 and 15 months and forecast the success of the artists listened for the first time in the remaining period. As shown in Table 5, our methodology correctly identifies *Hits* with high precision and recall with a relatively short training. In particular, *Hits&Flops* is able to maximize our target measures by learning the model on those artists that are listened for the first time during the first 9 months.

**Table 5:** *Hits&Flops* predictive performances when dealing with different temporal splits. Each column identifies a different split of observed 24 months: e.g., S6 stands for 6 months of training, 18 of test. In bold the best scores for PPV and TPR.

	*S*3	*S*6	*S*9	*S*12	*S*15
PPV	.79	.69	**.76**	.73	.67
NPV	.45	.57	.46	.54	.44
TPR	.13	.53	**.72**	.30	.83
SPC	.97	.72	.51	.88	.25

Once identified the best split strategy to learn our model, e.g., S9 in Table 5, we focus on the primary byproduct of our approach: the *Hitters* and *Floppers* sets. To characterize the stability of such sets, once fixed the splitting strategy, we learn *Hits&Flops* on several consecutive extractions obtained with a sliding window of fixed width. Fig 6 (right) shows, for both indicators, their similarity across time (continuous lines) and w.r.t. the first extraction (dashed lines). First of all, we observe that set stability is high: (i) consecutive *Hitter* sets do not change more than 40% and (ii) consecutive *Flopper* sets shown a Jaccard between 0.3 and 0.6. Conversely, we notice that, as expected, global stability (computed as the Jaccard among each new set and the first one) tends to decrease with time. In particular, after 9 months from model learning only 30% of original *Hitters* are still useful to predict successful artists. Our indicators are volatile and need to be updated incrementally to guarantee high predictive power, thus confirming what we observed while analyzing the distribution of active periods of *Hit-Savvy*.

At the end, who are the unexpected Flops among Last.fm artists? They are almost all the ones that receive huge media exposure (i.e., participants to music reality shows) that after their early explosion are not able to maintain the public engagement. Conversely, successful artists are the ones able to nurture their public by incrementally enter in the Last.fm scene with few individual tracks.

## 5 Related works

Our work is situated at the intersection of two intense research areas: (i) the identification and analysis of *Hit-Savvy* and *Innovators* and, (ii) the forecast of *Success*.

### Super-forecasters, Trend-Setter and innovators

The term super-forecaster has appeared only recently, primarily thanks to the seminal works of Tetlock [5, 6]. In [11] the authors theorized that the most successful forecasters did tend to have advanced degrees and a greater degree of general political knowledge. In [12] are studied the forecasts of 208 experts on the results of 15 treatments involving monetary and non-monetary motivators in a real-effort task: in such context, the authors identified *super-forecasters* among the non-experts who outperformed the experts w.r.t effort, confidence and revealed ability. As we have shown, super-forecaster can be seen as a very special subset of adopters: among the first studies on the adoptions of novel products [1, 2] hypothesized that the adoption trend distribution was likely to grow as an exponential toward an asymptote. In 1943, [3] theorised the existence of five categories of adopters: *Innovators*, *Early adopters*, *Early majority*, *Late majority* and *Laggards*. Rogers, in [4], revise such formulation defining adoption thresholds to separate the five categories: moving from the assumption of a normal distribution of adoption trends, he identifies the *innovators* with the first 2.5% of the adopters. As today, Rogers’ studies hold a predominant role within the study of information diffusion [13]: however, several models were proposed as alternative [14, 15]. In particular, as shown from the current work, three weakness of such model are often highlighted [16, 17]: (i) it is not justified empirically or analytically; (ii) it assumes that the adoption trend is normally distributed; (iii) the threshold is univocally defined for all the possible products. In recent years problem of identify *innovators* has lived a second youth: diffusion of Memes [18], social prominence analysis [19], discovery of innovation communities [20] as well as identification of *Trend-Setter* [21–24] are only a few of the network studies which exploit [25] or model [26] techniques aimed at identifying key users that are able to influence the diffusion of a product, content or idea over a social network. Although being related *Hit-Savvy* and *Trend-Setter* carry different semantics: where a *Hit-Savvy* is a passive actor that operates on the basis of its personal taste, a *Trend-Setter* is often defined as an active one that with his own choice aims to influence his peers. Indeed in a real-world scenario it is not easy to separate the former—which we can see as *taste-maker*—from the latter—that conversely are *taste-spotter* [27]. However, in the absence of a static/dynamic graph describing interactions among individuals and of an explicit/measurable index of causality/peer pressure, *Hit-Savvy* offer a less demanding definition than *Trend-Setters*, applicable, as in our cases, in a mean-field context.

### Science of success

One of the most intriguing challenges for data science is to forecast, given a partial observation, the future evolution of a phenomenon. Thanks to the growing availability of social data, sportive accomplishments [28–30], online habits [31] as well as, scientific publications [32–34] and professional accomplishments [35–37] new opportunities emerges for discovering the hidden patterns of success. Predictive models are also designed to facilitate the diffusion and adoption of innovations: machine learning literature has witnessed the increase of works in which are proposed context-specific models to suggest novel products to targeted adopters, as in [38]. Moreover, to identify success is necessary to be able to classify innovations relying on partial data. Finally, in [39] the authors propose a simple and effective method to provide uncertainty estimates for early classification of time series.

## 6 Conclusions

In this work we studied the *Hit-Savvy*, those special adopters having a propensity to adopt successful items. Our contribution was threefold: (i) firstly measure the intrinsic propensity of adopters toward Hit items, thus identifying the *Hit-Savvy*; (ii) we then analyze such special users on two different real-world datasets and observe their properties; (iii) finally we define a method that exploits *Hit-Savvy* to predict the future success of innovations given only partial observations of their adoptions.*Hits&Flops* does not depend on the success definition used to identify the *Hit-Savvy* and, our results showed that it is able to achieve high predictive performance thus being applicable to support marketing campaigns of novel items. Moreover, we validate the statistical significance of our approach against a null model concluding that the results obtained are due to the peculiar adoption behaviors of the identified *Hit-Savvy*. Given the obtained results we plan, as future works, to investigate if a specialization of our approach in a given context (i.e., learn it for a well-defined music genre or product segment) can lead to even more interesting performances. Finally, it would be interesting to understand if the social relationships among adopters, as well as exogenous definitions of success, can be used to characterize *Hit-Savvy* better. Moreover, leveraging detailed information on adopters interactions could allow a data-driven support for the study of the relations among *Hit-Savvy* and *Trend-Setters*.

## Supporting information

S1 Fig Coop trends analysis. Cluster medoids identified by k-means with DTW. Also for this dataset, the medoids are well-separated and describe characteristic shapes: one expressing a sudden drop of the adoption rate (identified by a dashed blue line), the other capturing an expanding trend (identified by a red line).
(PDF)

S2 Fig Coop trend volumes. Comparison of volumes of expanding and contracting trends. The results show the same tendency of Last.fm dataset: items having expanding trends tend, on average, to have a broader diffusion than the others.
(PDF)

S3 Fig Coop predictive accuracy. Predictive accuracy varying the observation period for the Coop dataset. We notice that the main effect of a protracted observation is to introduce variability on Recall and Specificity; with more observations, we are likely to identify a higher percentage of *Hits* preserving a high PPV and NPV. For this specific case study, the optimal observation window can be reasonably fixed at 4 weeks since, with such settings, we can observe the best predictive power.
(PDF)
