# Supplementary figures and images for: Forecasting success via early adoptions analysis: A data-driven study
Source: PLoS One. 2017 Dec 7;12(12):e0189096. doi: 10.1371/journal.pone.0189096 (PMC5720712; doi:10.1371/journal.pone.0189096)

## COOP Clustering

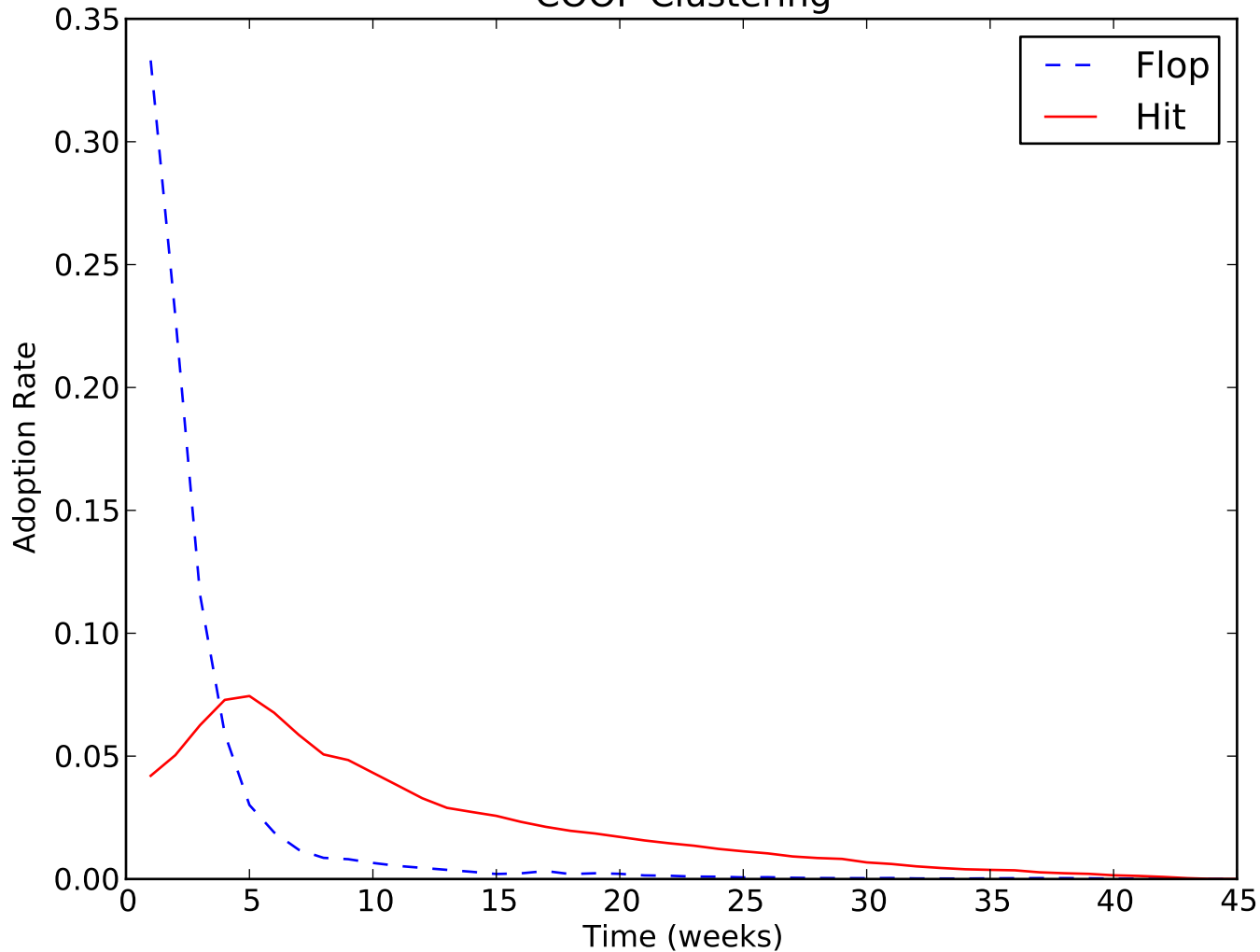

Supplement: S1 Fig — Cluster medoids identified by k-means with DTW. Also for this dataset, the medoids are well-separated and describe characteristic shapes: one expressing a sudden drop of the adoption rate (identified by a dashed blue line), the other capturing an expanding trend (identified by a red line). (PDF) [file pone.0189096.s001.pdf]

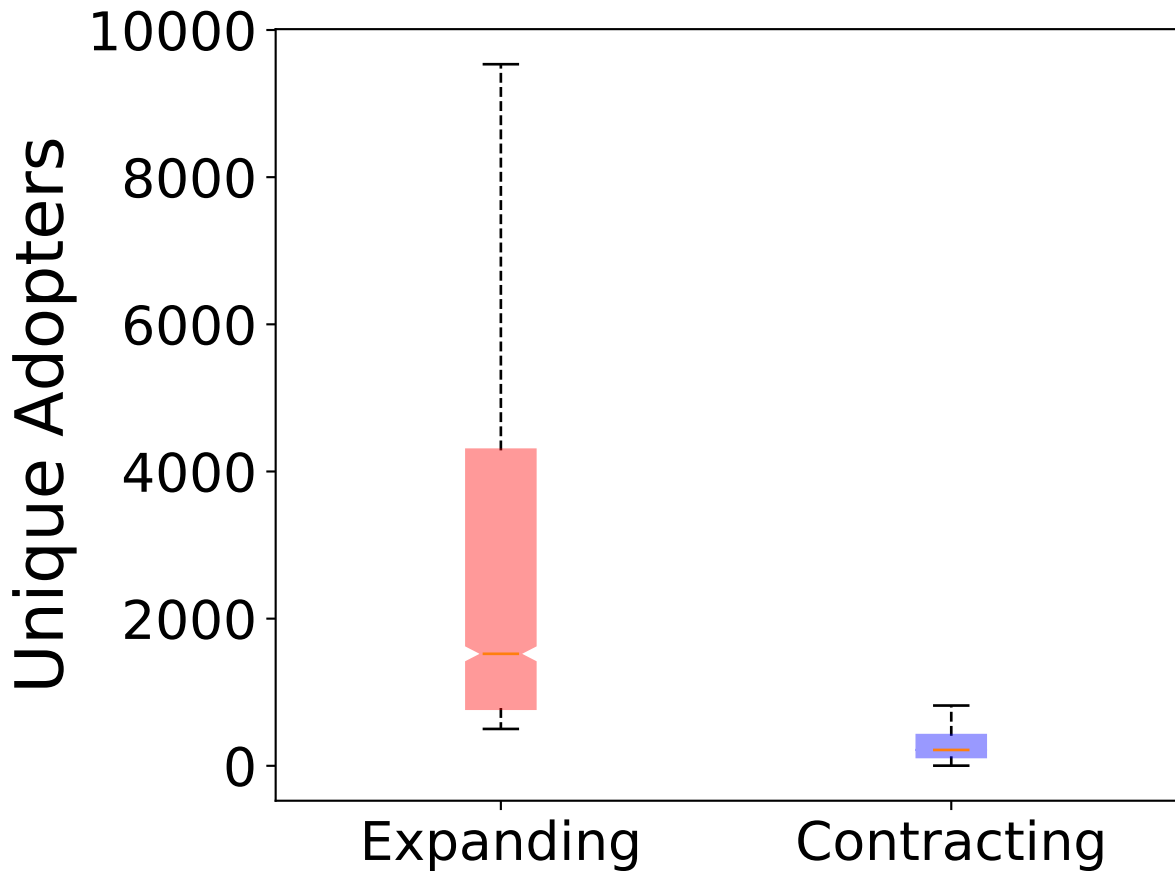

Supplement: S2 Fig — Comparison of volumes of expanding and contracting trends. The results show the same tendency of Last.fm dataset: items having expanding trends tend, on average, to have a broader diffusion than the others. (PDF) [file pone.0189096.s002.pdf]

## COOP timescale

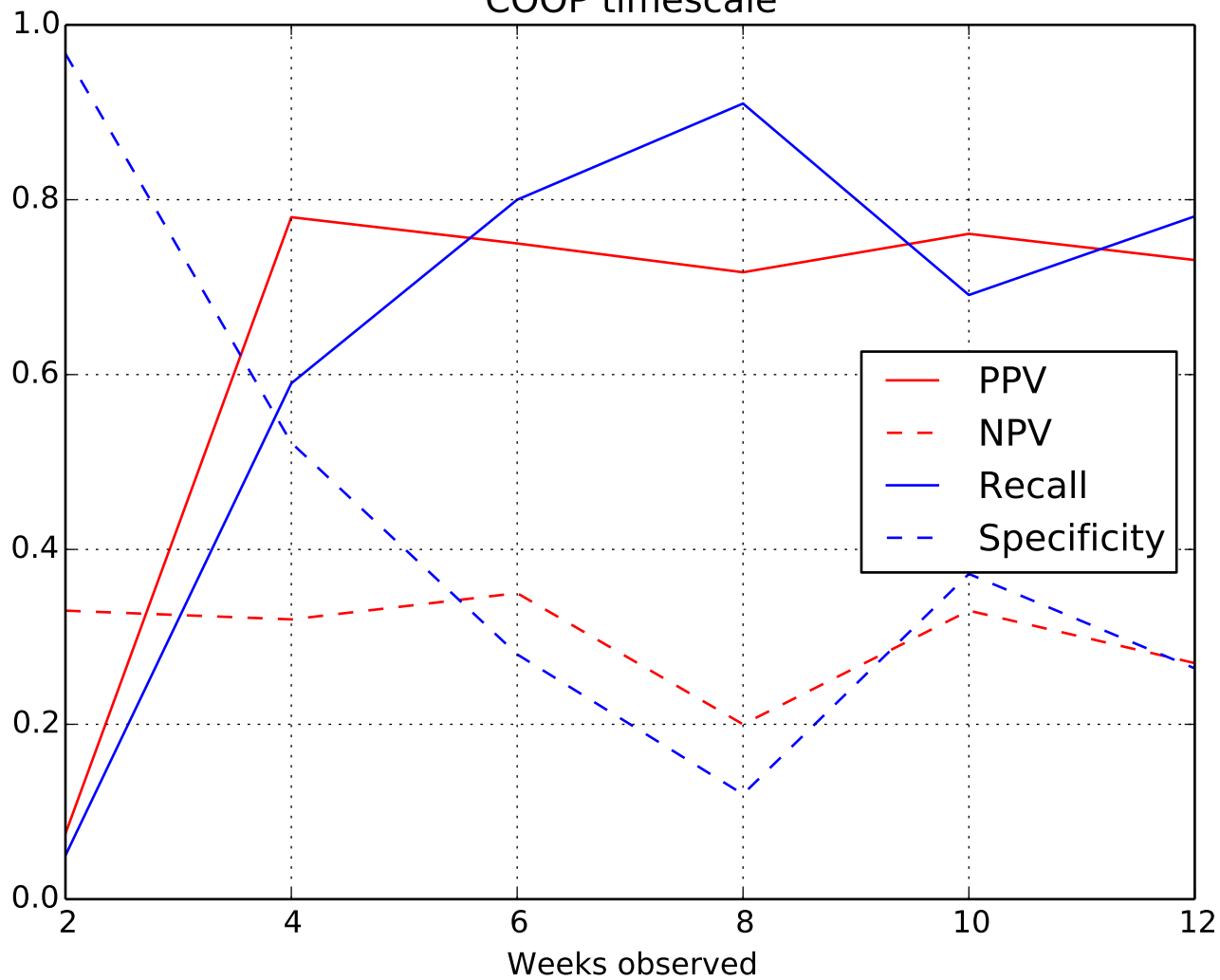

Supplement: S3 Fig — Predictive accuracy varying the observation period for the Coop dataset. We notice that the main effect of a protracted observation is to introduce variability on Recall and Specificity; with more observations, we are likely to identify a higher percentage of Hits preserving a high PPV and NPV. For this specific case study, the optimal observation window can be reasonably fixed at 4 weeks since, with such settings, we can observe the best predictive power. (PDF) [file pone.0189096.s003.pdf]
